# iCAT: diagnostic assessment tool of immunological history using high-throughput T-cell receptor sequencing

**DOI:** 10.12688/f1000research.27214.1

**Published:** 2021-02-03

**Authors:** Ahmad Rajeh, Kyle Wolf, Courtney Schiebout, Nabeel Sait, Tim Kosfeld, Richard J. DiPaolo, Tae-Hyuk Ahn

**Affiliations:** 1 Program in Bioinformatics and Computational Biology, Saint Louis University, St. Louis, MO, 63103, USA; 2 Molecular Microbiology and Immunology, Saint Louis University School of Medicine, St. Louis, MO, 63104, USA; 3 Computer Science, Saint Louis University, St. Louis, MO, 63103, USA

**Keywords:** T-cell receptor sequencing, diagnostic classification, R-package, biomarkers

## Abstract

The pathogen exposure history of an individual is recorded in their T-cell repertoire and can be accessed through the study of T-cell receptors (TCRs) if the tools to identify them were available. For each T-cell, the TCR loci undergoes genetic rearrangement that creates a unique DNA sequence. In theory these unique sequences can be used as biomarkers for tracking T-cell responses and cataloging immunological history. We developed the immune Cell Analysis Tool (iCAT), an R software package that analyzes TCR sequencing data from exposed (positive) and unexposed (negative) samples to identify TCR sequences statistically associated with positive samples. The presence and absence of associated sequences in samples trains a classifier to diagnose pathogen-specific exposure. We demonstrate the high accuracy of iCAT by testing on three TCR sequencing datasets. First, iCAT successfully diagnosed smallpox vaccinated versus naïve samples in an independent cohort of mice with 95% accuracy. Second, iCAT displayed 100% accuracy classifying naïve and monkeypox vaccinated mice.

Finally, we demonstrate the use of iCAT on human samples before and after exposure to SARS-CoV-2, the virus behind the COVID-19 global pandemic. We were able to correctly classify the exposed samples with perfect accuracy. These experimental results show that iCAT capitalizes on the power of TCR sequencing to simplify infection diagnostics. iCAT provides the option of a graphical, user-friendly interface on top of usual R interface allowing it to reach a wider audience.

## Introduction

T- and B-cell responses are responsible for long-lasting immune memory responses to infectious agents, such as bacteria and viruses. Expansion of pathogen specific T-cells provide us with a robust resource for understanding whether an individual has been infected with a pathogen. The T-cell receptor (TCR), located on the surface of T-cells, is responsible for recognizing pathogen-specific peptides, leading to immune response and development of protective immune memory ^1^. During T-cell development, the loci that encode TCRs *α* and *β*-chains are rearranged by recombination of the variable (TCRV), diversity (TCRD), and joining (TCRJ) gene segments, encoding the complementary determining region 3 (CDR3) ^2^. These genetic rearrangement events result in a high degree of diversity in the CDR3 regions of individual TCR loci ^3^.

During an infection or vaccination, T-cells that carry receptors recognizing pathogen associated peptides become activated and undergo rapid clonal expansion. The clonally expanded T-cells carry the same unique TCR rearrangement and a portion remain in circulation long after the pathogen has been cleared to provide long-lived immunological memory. The persistence of memory T-cells in circulation make the genetically rearranged TCR loci a stable biomarker documenting an individual’s immunological history. To utilize the diverse TCR repertoire as a potential biomarker for specific pathogen exposure, pathogen-specific TCR sequences common to different individuals exposed to the same pathogen need to be identified. This poses significant challenges given the diversity and magnitude of the TCR repertoire. On average, ∼ 10 ^7^ unique TCR *β* chains can be identified from the ∼ 10 ^12^ circulating T-cells present in a healthy human adult ^4^. A healthy human adult can have 10 ^18^ mathematically possible TCR recombinations resulting from the genetic rearrangement ^4^^,^^5^. The potential diversity of the repertoire coupled with the limited number of T-cells present in individuals makes identifying identical TCR sequences among multiple individuals exceptionally challenging. However, by analyzing the large and diverse TCR repertoire using high-throughput TCR *β* sequencing, it is possible to identify pathogen specific TCRs shared among different individuals exposed to the same infectious agent ^6^.

Recently, high-throughput next-generation sequencing (NGS) techniques were employed to analyze the diverse immune cell repertoire ^7‐9^. Additionally, recent publications described an analytical approach for computationally identifying common/public TCR sequences ^5^^,^^9^^,^^10^. However, analyses of the TCR repertoire for diagnostic purposes have remained largely resource- and time-intensive efforts.

Utilizing the diagnostic methodologies described in Wolf, et al. (2018) ^5^, we have developed an R package with a user-friendly interface, the immune Cell Analysis Tool ( iCAT), to identify TCR sequences statistically associated with pathogen exposure and to distinguish infected from non-infected samples. By providing an interactive interface through iCAT, sample exposure can be assessed and predicted conveniently without requiring command-line skills. iCAT has an ability to classify target-associated receptor sequences (TARSs) and diagnose exposure with a high accuracy.

## Methods

### Implementation

**Figure 1.**
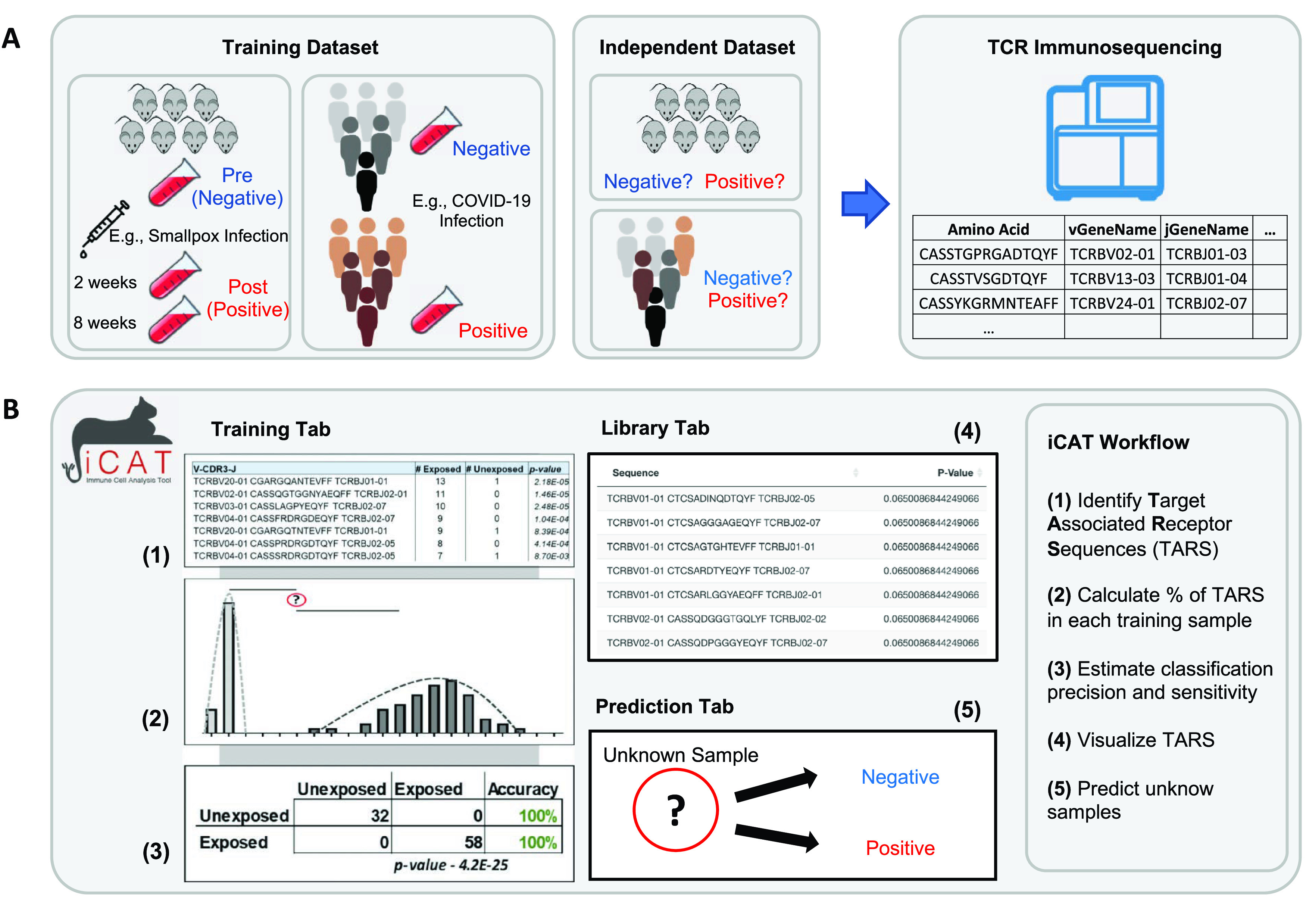
Workflow for TCR repertoire sequencing and diagnostic assessment of prior antigen exposure using iCAT. A) Flow chart depicting the purification of genomic DNA from blood samples and the production of TCR repertoires after TCR-specific amplification and sequencing. B) Visual representation of the iCAT methodology.

We developed iCAT, an R package utilizing high-throughput TCR sequencing data to analyze TCR sequences and to diagnose infection in a user-friendly format ( Figure 1). iCAT provides both a graphical user interface (GUI) in the form of a web-application utilizing R-Shiny ^11^ and a command-line R interface for batch processing of large-scale data. The simplest method to install iCAT on a system is directly from GitHub using devtools and install_github:

install.packages("devtools")
devtools::install_github("BioHPC/iCAT")



In addition to shiny, iCAT also uses shinyjs, data.table, ggplot2, DT, hash, and magrittr. Required packages will be installed through the install_github step. Alternatively, users can clone or download the repository from GitHub and run devtools::install("iCAT/").

**Figure 2.**
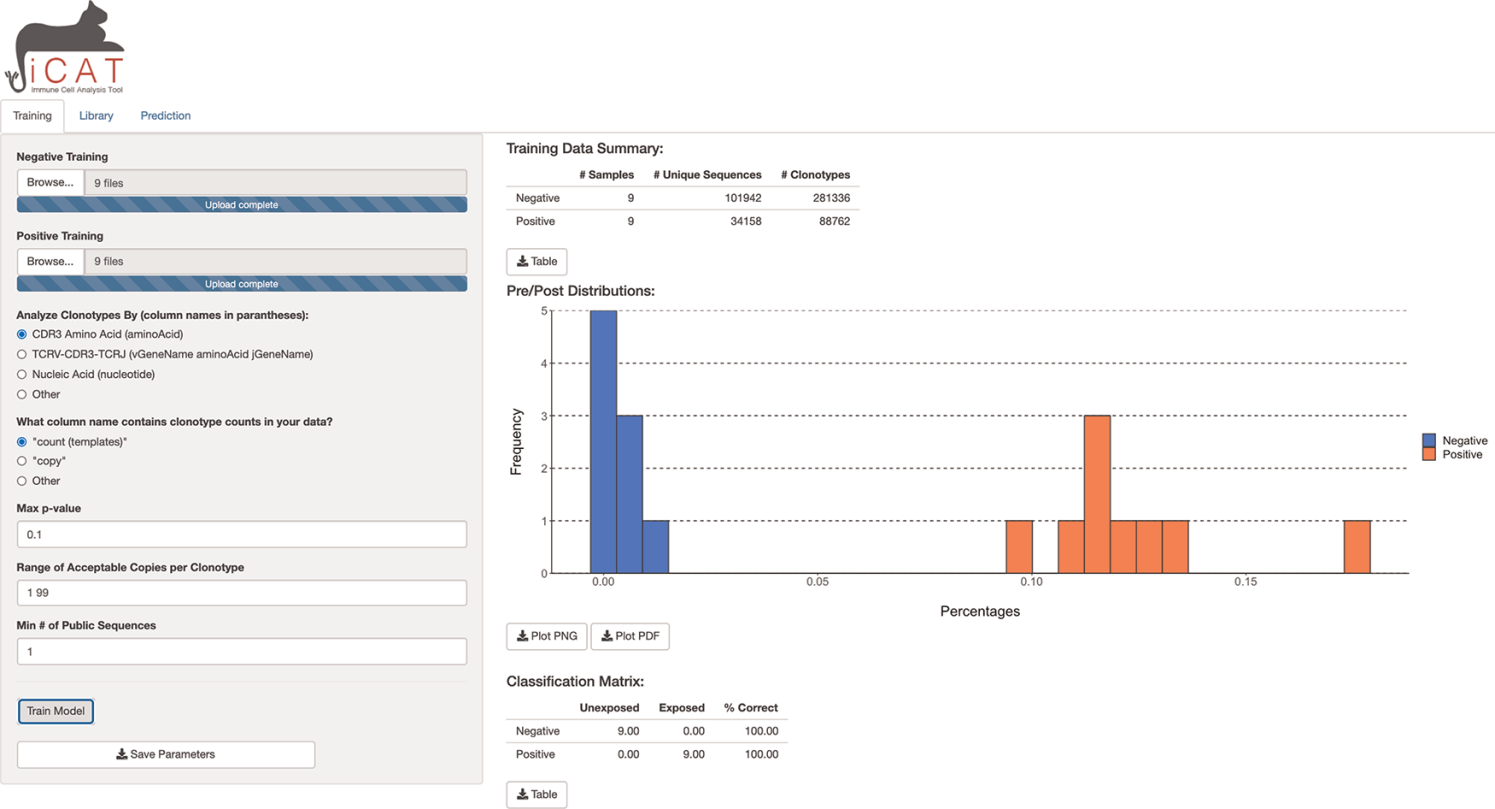
iCAT Training tab. After samples are uploaded, clicking “Training” will start training to select features for the diagnostic classifier from the negative and positive samples.

A user can upload multiple TCR sequence repertoires from negative (control) and positive (experimental) cohorts. iCAT accepts tab-delimited files with the size limit of 10 gigabytes per file with multiple options to define TCR clonotypes within samples. The iCAT shiny app has three tabs, separating major functionalities: Training, Library, and Prediction. Under the “Training” tab, clicking ‘Train Model’ will start the pipeline to statistically identify a subset of TARSs that will act as feature selections for training the diagnostic classifier, diagnosing samples as either negative or positive. Upon training, iCAT’s main tab provides a table summary of the data, a figure shows the distribution of TARSs between the positive and negative samples, and a classification matrix predicting the exposure status of samples used in the training data ( Figure 2). All figures and tables can be downloaded to the user’s machine. A progress bar will show on the bottom-right corner to update on the status of training.

**Figure 3.**
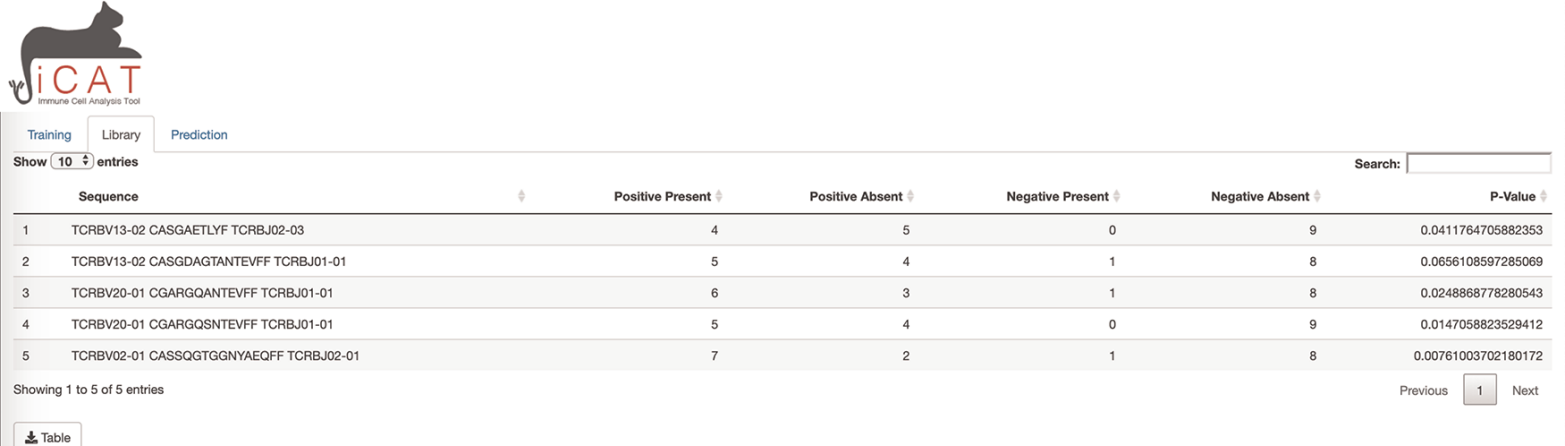
iCAT Library tab. The library tab shows a table of target-associated receptor sequences (TARS).

A separate tab, “Library”, is unlocked upon training and shows a table where each row describes a TARS and its presence in the positive and negative samples. All tables and figures are supplemented with a custom button for easy download ( Figure 3).

**Figure 4.**
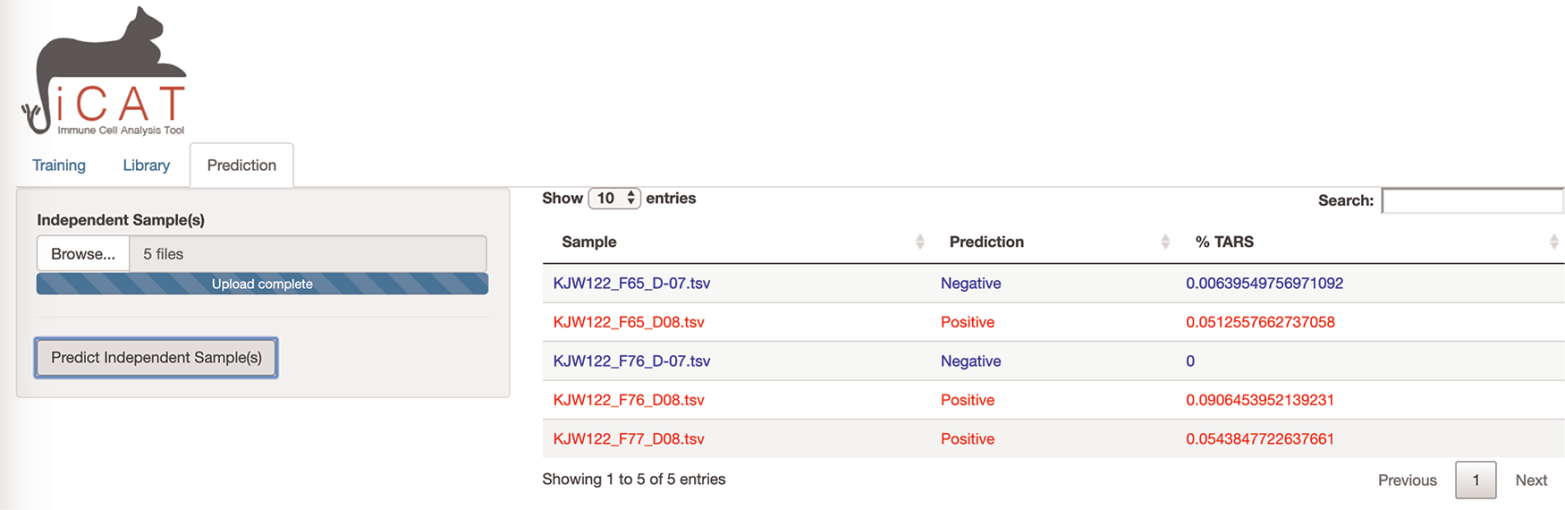
iCAT Prediction tab. The prediction tab allows the user to upload one or more independent TCR-sequencing samples for classification.

The third tab of iCAT, “Prediction”, also unlocks after training and allows the user to upload one or more independent TCR-sequencing samples for classification ( Figure 4).

### Operation

Samples, such as from blood or lymph tissue, are collected and genetic material is purified. TCR sequences present in the sample are selectively amplified and then sequenced ( Figure 1A). The first step of iCAT is the “ Training” step. A user should provide multiple negative training samples (naïve, unexposed, uninfected, etc.) using the Browse button. Then, repeat the step for positive training samples (exposed, infected, vaccinated, etc.). The user should select the type of training feature. iCAT provides three options: (1) CDR3 Amino Acid Sequence (TCRs will need the same CDR3 region to be called ”Identical”), (2) TCRV-CDR3-TCRJ (TCRs will need the same TCRBV segment, CDR3 region, and TCRJ segment to be called ”Identical”), (3) Nucleic Acid (DNA) (TCRs will need the exact same DNA rearrangements/sequence across TCRBV, CDR3, and TCRJ). Selecting TCRV-CDR3-TCRJ is recommended as a balance between sensitivity and specificity and this option has been used for all the use cases in this paper. In addition, users can customize the range acceptable of copies per clonotype, and the minimum threshold of public sequences, which determines the minimum samples a TCR sequence must be observed in to be considered for analysis.

One important option of this “ Training” tab is Max p-value (default: 0.1), which determines the minimal degree of statistical significance that iCAT will accept as being potentially ”associated” with the positive group. The statistical methodology of iCAT is based on identifying a subset of TARSs that informs classification ^5^. TCR sequences significantly associated with positive samples as opposed to negative samples are identified by performing a one-tailed Fisher’s exact test. iCAT determines the optimal p-value cutoff to generate the TARS library based on the idea of coverage ratio. To determine an optimal p-value threshold for identifying vaccine-associated TCR *β* sequences, we applied a heuristic test that selected the optimal p-value threshold based on the ”coverage” provided by the library for both vaccinated ( *C*_*v*_) and naïve samples ( *C*_*n*_) ^5^. “Coverage” is defined as the summation of the number of samples containing each VATS divided by the number of samples. In the equations below, *x*_*i*_ denotes the number of vaccinated samples a single TCR *β* is identified and *n*_*v*_ represents the number of positive samples in the training data. *y*_*i*_ also denotes for naïve samples and *n*_*n*_ represents the number of naïve samples.

$$C_{v}=\frac{\sum_{i=1}x_{i}}{n_{v}},C_{n}=\frac{\sum_{i=1}y_{i}}{n_{n}}.$$ (1)

The ratio of *C*_*v*_ to *C*_*n*_ is determined for each p-value. The p-value with the largest *C*_*v*_: *C*_*n*_ ratio and offers significant coverage to distinguish (v)accinated from (n)aïve samples was chosen.

For the classification of vaccinated and naïve samples, iCAT calculates the percentage of TARS (% TARS) present in a sample. The % TARS for each sample in the training data is compared against the % TARS normal distribution for each group to predict if each sample is ”positive” or ”negative”, determined by which group a sample is more closely associated with ^5^. Such a normal distribution has been adopted to calculate the distance of a sample to the mean. In detail, the normal distributions for the naïve and vaccinated populations in our training data were calculated based on a function of the difference between a single sample value ( *x*) and the mean of a set of data ( *μ*) over the standard deviation of that set of data ( *σ*) utilizing a correction constant to remove negative selection bias of the naïve training sample data from the below equation.

$$f(x|\mu ,\sigma^{2})=(\frac{1}{\sqrt{2\pi \sigma^{2}}})\exp^{-\frac{{\left(x-\mu \right)}^{2}}{2\sigma^{2}}}.$$ (2)

If the value is bigger, we can conclude that the sample is more associated to the the training group. By comparing a sample against the normal distribution of vaccinated and naïve training groups, we can determine which group a sample is more statistically associated with. A progress bar will show on the bottom-right corner to update on the status of training. After finishing, the “ Training” tab will show some exploratory tables and a figure regarding the training data and the model built, which can all be downloaded to the user’s machine easily. In addition, the “ Library” and “ Prediction” tabs will unlock.

The “ Library” tab displays a table consisting of the TARS, determined to be statistically associated with exposure to the target/agent/pathogen ( Figure 3). The table displays each sequence, number of positive and negative training samples the sequence is present in/absent from, and how statistically associated the sequence is to the positive training data (p-value). The table can be downloaded to the user’s computer for further analysis ( Figure 3).

To allow the diagnostic classifier to test independent cohorts of samples, a third tab in iCAT, “ Prediction” tab, also unlocks after training and allows the user to upload one or more independent TCR-sequencing samples for classification using the parameters generated by the training data ( Figure 4). The “ Prediction” tab allows the user to diagnose unknown samples (e.g. not included in the previous training data) for classification as “Positive” or “Negative” and determining the accuracy of the diagnostic assay. Use the Browse button to upload such independent samples for prediction. Multiple samples can be uploaded simultaneously. Click Predict Independent Sample will analyze the dataset. A table will appear after analysis is complete. The table displays sample names along with the prediction ”Positive” (red) or ”Negative” (blue), and displays the %TARS that is the percent of individual sequences from the sample that are included in the TARS library. The prediction results can be downloaded as a table.

iCAT requires R 3.4.0 or upper and can be run on any operating system with common specifications (1 GB disk space, 4 GB memory, and multicore CPU is recommended).

## Use cases

To evaluate the efficacy of iCAT, we used three TCR sequencing data sets that are publicly available. The first viral data set consists of 148 training and 20 independent mouse samples. The training set has 32 mouse pre-treatment naïve group and 116 vaccinated samples, each cohort inoculated intranasally with the ACAM2000 smallpox vaccine. The second viral data set consists of 133 (27 negative and 96 positive) training and 15 (5 negative and 20 positive) monkeypox virus. The two mouse datasets are publicly available from https://doi.org/10.17632/cf92gt44zf.1. The third data set is human TCR samples exposed to the novel SARS-CoV- 2 virus which is the cause of the ongoing COVID-19 global pandemic ^12^. The sample size is small (two cohorts), but those two cohorts’ TCR repertoires were obtained for other projects one and two years prior to infection. Therefore, this negative and positive SARS-CoV-2 human TCR sequencing data set from same cohorts can be a great example to show the great potential of iCAT as a diagnostic assay. This data is available at https://doi.org/10.5281/zenodo.3835956.

### Use case 1: smallpox mouse data

32 pre-exposure (naïve) samples were analyzed, which included 2,049,383 unique TCR sequences (clonotypes). We setup iCAT options to analyze by either the CDR3 amino acid sequence or the V-gene and J-gene names. 714,522 amino acid sequence-gene name combinations were found in the naïve samples. 58 samples taken 2- and 8-weeks post-vaccination for smallpox were analyzed, which included 1,581,619 clonotypes and 573,612 unique amino acid sequence-gene name combinations. After training, iCAT accurately generated the same virus-associated TCR library (314 TCR sequences) identified in Wolf, et al., 2018 ^5^. From the training data, iCAT correctly classified 32 of 32 naïve samples as ”unexposed” and 58 of 58 vaccinated samples as ”exposed” (100% accuracy). We utilized TCR-sequencing files from 10 mice pre- and post-smallpox vaccination that were not involved in the training of the diagnostic classifier to act as independent cohorts to test the diagnostic accuracy of the iCAT generated classifier. The classification results are displayed in the “Prediction” tab and can be downloaded as a .txt file. From a total of 20 samples, 90% of pre-vaccination samples (9 of 10) were correctly classified as “negative” and 100% of samples post-smallpox vaccination (10 of 10) were classified as ”positive”. Overall, this data displays that the iCAT platform computationally identifies target-associated public TCRs, utilized to train a diagnostic classifier capable of distinguishing between exposed and unexposed samples with a high degree of accuracy.

### Use case 2: monkeypox mouse data

We tested iCAT using another TCR-sequencing mouse dataset which included 27 naïve samples and 48 samples 2- and 8-weeks post infection with monkeypox. We chose to analyze based on CDR3 amino acid sequence in addition to V-gene and J-gene names. The p-value cutoff was set to 0.1 and the minimum number of public sequences was set to 1. Those parameters produced the best separation experimentally. Naïve samples included 1,772,085 clonotypes and 630,381 unique amino acid sequence-gene name combinations. Exposed samples included 1,070,615 clonotypes and 382,906 unique amino acid sequence-gene name combinations. iCAT correctly classified this training data with 100% accuracy. When tested on an independent monkey pox data set – set up by excluding 5 samples from the naïve group and 10 samples from the exposed group pre-training – iCAT correctly classified the 5 naïve samples as negative and the 10 exposed samples as positive. Thus, we demonstrated a 100% classification accuracy using iCAT on this monkeypox dataset.

### Use case 3: human SARS-CoV-2 data

We further tested iCAT on TCR-sequencing data from two human individuals exposed to the novel SARS-CoV-2 virus ^12^. Data included 4 naïve samples from 2018 and 2019, and 4 samples collected 15- and 30-days post-infection. iCAT was configured to analyze by CDR3 amino acid sequence only. The p-value cutoff was set to 0.1 and the minimum number of public sequences was set to 1. The naïve data included 2,935,893 clonotypes and 1,120,606 unique CDR3 amino acid sequences. The exposed data included 1,987,608 clonotypes and 541,111 unique amino acid sequences. iCAT achieved a perfect classification accuracy on the training data, correctly assigning the 4 naïve and 4 exposed samples. Further, iCAT correctly classified 4 independent exposed samples as positive. This demonstrates the wide utility of iCAT and the methodology it implements.

## Conclusions

In this article, we have presented iCAT, a powerful software tool for determining pathogen exposure through TCR sequencing data. It has significant clinical applications in disease diagnosis, surveillance, as well as for determining potential vaccine efficacy. Once data interpretation is fully automated, the TCR sequencing analysis and other types of NGS will likely become a standard tool for diagnosis and management of disease. Our current datasets are from pre- and post- exposure to viruses, and serve as a proof of principle that TCR sequencing analysis can be utilized to identify individuals exposed to infectious agents or vaccines with great accuracy, speed, and accessibility. We demonstrated the use of iCAT for accurately detecting exposure to the SARS-CoV-2, the virus behind COVID-19. Although this use case was based on a few number of samples, it shows the immense potential of our software the utilization of TCRs as a biomarker. This type of analysis may be used to distinguish between two different but highly related infections, such as Zika virus and Dengue, which is one of the global concerns considering Zika virus’s association with fetal complications in infected pregnant women, and current laboratory testing cannot distinguish between the two. Parallel endeavors in our group show promising results in identifying virus-associated TCR sequences uniquely associated with a prior Zika versus Dengue virus infection in mice using iCAT. Further, the iCAT platform may prove useful for diagnosing individuals in the early stages of autoimmunity, by identifying auto-reactive TCRs before symptoms and significant tissue damage occurs. Earlier diagnosis may allow for preventative measures, better treatment, and better outcomes. Broadly, our approach can be used to diagnose autoimmune disease and possibly immune responses to cancer before or after immunotherapy.

## Data availability

Mendeley Data: Identifying and Tracking Low Frequency Virus-Specific TCR Clonotypes Using High-Throughput Sequencing, https://doi.org/10.17632/cf92gt44zf.1 ^13^.

This project contains the raw sequencing data from HLA-A2 transgenic mice before and after infection with either the ACAM2000 smallpox virus or highly releated monkeypox virus.

Zenodo: Longitudinal high-throughput TCR repertoire profiling reveals the dynamics of T cell memory formation after mild COVID-19 infection, https://doi.org/10.5281/zenodo.3835956 ^14^.

This project contains the third human TCR samples exposed to the novel SARS-CoV-2 virus is available from zenodo in mixcr format.

Data are available under the terms of the Creative Commons Attribution 4.0 International license (CC-BY 4.0).

## Software availability

Source code available from: https://github.com/BioHPC/iCAT.

Archived sourced code as at time of publication: http://doi.org/10.5281/zenodo.4436485 ^15^.

License: MIT
