# Supplementary material for: iCAT: diagnostic assessment tool of immunological history using high-throughput T-cell receptor sequencing
Source: F1000Res. 2021 Jun 29;10:65. Originally published 2021 Feb 3. [Version 2] doi: 10.12688/f1000research.27214.2 (PMC8276190; doi:10.12688/f1000research.27214.2)
Supplement: Peer review history

# Peer review: Reviewer response for version 2

**DOI:** 10.5256/f1000research.57705.r88494

**Published:** 2021-07-12

**Authors:** Se-Ran Jun

**Affiliations:** 1 Department of Biomedical Informatics, University of Arkansas for Medical Sciences, Little Rock, AR, USA

Thank you so much for the improved manuscript. I have minor comments:

- Please add a sentence(s) on how you calculated uncertainty.
- Please check the following sentence: "The p-value with the largest *C _v_*: *C _n_* ratio and offers significant coverage to distinguish vaccinated ( *v*) from naïve ( *n*) samples was chosen."
- Is the following sentence correct: "If the value is bigger, we can conclude that the sample is more associated with the training group."?

Are the conclusions about the tool and its performance adequately supported by the findings presented in the article?

Yes

Is the rationale for developing the new software tool clearly explained?

Partly

Is the description of the software tool technically sound?

Partly

Are sufficient details of the code, methods and analysis (if applicable) provided to allow replication of the software development and its use by others?

Partly

Is sufficient information provided to allow interpretation of the expected output datasets and any results generated using the tool?

Partly

Reviewer Expertise:

Genomic Epidemiology, Microbiome Epidemiology, Multiomics

I confirm that I have read this submission and believe that I have an appropriate level of expertise to confirm that it is of an acceptable scientific standard.

# Peer review: Reviewer response for version 2

**DOI:** 10.5256/f1000research.57705.r88495

**Published:** 2021-07-06

**Authors:** Shuo-Wang Qiao, Ying Yao

**Affiliations:** 1 Department of Immunology, Oslo University Hospital, Rikshospitalet, University of Oslo, Oslo, Norway; 2 Department of Immunology, University of Oslo, Oslo, Norway

No further comments.

Are the conclusions about the tool and its performance adequately supported by the findings presented in the article?

Yes

Is the rationale for developing the new software tool clearly explained?

Yes

Is the description of the software tool technically sound?

Partly

Are sufficient details of the code, methods and analysis (if applicable) provided to allow replication of the software development and its use by others?

No

Is sufficient information provided to allow interpretation of the expected output datasets and any results generated using the tool?

Yes

Reviewer Expertise:

Immunology, T cells, TCR, single-cell receptor sequencing,

We confirm that we have read this submission and believe that we have an appropriate level of expertise to confirm that it is of an acceptable scientific standard.

# Peer review: Reviewer response for version 1

**DOI:** 10.5256/f1000research.30069.r82161

**Published:** 2021-04-13

**Authors:** Se-Ran Jun

**Affiliations:** 1 Department of Biomedical Informatics, University of Arkansas for Medical Sciences, Little Rock, AR, USA

This article is a valuable resource for T-cell receptor (TCR) sequencing data-based infection diagnostics. The authors provided a R software package named iCAT which provides a step-by-step analysis of TCR sequencing data to predict if individual subjects are infected or not. First, iCAT takes positive and negative pathogen-specific TCR datasets to estimate parameters involved in in model. Second, iCAT provides the Library tab which summarizes the target associated receptors sequences with p-values. Third, iCAT provides prediction tab which takes test TCR datasets and make diagnostic decision. I enjoyed reading this article and github tutorial.

**Major comments:**

 It seems like that the performance of this tool does depend on the number of samples.  Please provide discussion or guideline on the number of negative and positive samples relating to the performance.

 It seems like that this is the first tool available for TCR data-based diagnostic tool. Please clarify if this is the first tool which uses TCR sequencing data for the purpose of diagnostic. If this is not the first tool, then comparison results with other tools should be included.

**Minor comments:**

 First, I have a question for clarification purpose. Before TCR sequencing data is uploaded, does TCR sequencing data need any preprocessing step to make input compatible with iCAT?

 Install.packages(“devtools”) does not work with R4.0.3 on Mac OS X. The error I got is as follows:

 Downloading GitHub repo BioHPC/iCAT@HEAD

 Error in utils::download.file(url, path, method = method, quiet = quiet,  :

 download from 'https://api.github.com/repos/BioHPC/iCAT/tarball/HEAD' failed

 Each input file is limited to 10Gigabyte in size. Where does this limitation come from?

 I downloaded one of test cases which contains many files. It was not easy to figure out which files to be uploaded. It would be nice that this R package provides example datasets along with the package so that they can be played in R directly.

 There is an option named ‘Other’ for ‘analyze clonotypes by (column names in parantheses)’. What is other?

 Please include information of computational time for training and prediction steps for each case.

 Although feature from an option of ‘TCRV-CDR3-TCRJ’ is recommended, please include the performance for other features for each test case which could help readers understand and compare features.

 What does TCRBV stand for?

 What does VATS stand for?

 A correction constant is not shown in equation (2).

 Whole genome sequences can distinguish DENGU from ZIKE with 100% accuracy. For the sentence ‘current laboratory testing cannot distinguish between the two’, are you excluding whole genome sequencing data?

 Is it possible to make change range of acceptable into something like 1:99 or 1~99 instead of 1 99 to indicate range?

 Does the max p-value change automatically according to the training data provided? Or should it be changed manually based on the selected one by Fisher’s t-test?

 Please revise typo in Figure 1(B)

 Please revise typo with ‘the minimum samples’

 Please revise the following sentences:

 - The ratio of Cv to Cn is determined for each p-value. The p-value with the largest Cv:Cn ratio and offers significant coverage to distinguish (v)accinated from (n)aïve samples was chosen.

 - iCAT was configured to analyze by CDR3 amino acid sequence only

 If the value is bigger, we can conclude that the sample is more associated to the the training group. Is this a correct sentence?

 From the training data, iCAT correctly classified 32 of 32 naïve samples as ”unexposed” and 58 of 58 vaccinated samples as ”exposed” (100% accuracy). Is this a correct sentence?

Are the conclusions about the tool and its performance adequately supported by the findings presented in the article?

Yes

Is the rationale for developing the new software tool clearly explained?

Partly

Is the description of the software tool technically sound?

Partly

Are sufficient details of the code, methods and analysis (if applicable) provided to allow replication of the software development and its use by others?

Partly

Is sufficient information provided to allow interpretation of the expected output datasets and any results generated using the tool?

Partly

Reviewer Expertise:

Genomic epidemiology, Multiomics

I confirm that I have read this submission and believe that I have an appropriate level of expertise to confirm that it is of an acceptable scientific standard, however I have significant reservations, as outlined above.

# Response

**Published:** 2021-06-10

**Authors:** Tae-Hyuk Ahn

**Affiliations:** Saint Louis University, USA

**Major comments:**

 1. It seems like that the performance of this tool does depend on the number of samples.  Please provide discussion or guideline on the number of negative and positive samples relating to the performance.

 Response: We really appreciate all the great and thoughtful comments from the reviewer. The sample size is of course very important in determining the power and accuracy of the diagnostic.  We have generated positive and negative datasets of various sample sizes to determine the impact of sample numbers on accuracy.  The conclusion was that the number of samples required to generate an accurate diagnosis is determined by the nature of the samples. For example, a dataset from Zika infected mice produced a small but conserved virus-specific TCR repertoire due to the small number of immunogenic epitopes (Hassert and Wolf et. al. 2020) ; this allowed for the generation of a powerful diagnostic assay using relatively small numbers of samples.  Comparatively, using a dataset from mice before and after smallpox vaccination resulted in a large pool of virus-specific TCRs due to a large number of potential epitopes; the less focused response required a greater number of samples in each group to generate sufficient coverage and diagnostic accuracy (Wolf et. al. 2018).

 2. It seems like that this is the first tool available for TCR data-based diagnostic tool. Please clarify if this is the first tool which uses TCR sequencing data for the purpose of diagnostic. If this is not the first tool, then comparison results with other tools should be included.

 Response: To our limited knowledge, this is the first publicly available tool for diagnostic classification using TCR sequencing data.

**Minor comments:**

 1. First, I have a question for clarification purpose. Before TCR sequencing data is uploaded, does TCR sequencing data need any preprocessing step to make input compatible with iCAT?

 Response: iCAT takes in TCR data in tab-delimited clonotype abundance tables. These tables are the result of mapping of reads from Variable, Diversity, and Joining segments then the assembly of clonotypes. iCAT does not perform these steps so they may be considered pre-processing. Clonotype tables are provided through the immunoSEQ assay from Adaptive Technologies, which is the most commonly used immunosequencing pipeline in recently published studies. While iCAT input-parsing was built around immunoSEQ’s format, it also has a flexible interface that allows handling of different formats with different user-selected column names.

 2. Install.packages(“devtools”) does not work with R4.0.3 on Mac OS X. The error I got is as follows:

 This issue is resolved and updated on iCAT.

 3. Each input file is limited to 10Gigabyte in size. Where does this limitation come from?

 Response: This is an arbitrary limitation that we coded into the tool’s interface. It is not a required limitation for any of the underlying functions in iCAT. The reason we put this size limit in place is because one of our future goals with iCAT is to host it on a website where users can upload their data for analysis, and thus data size can become a limiting factor.

 4. I downloaded one of test cases which contains many files. It was not easy to figure out which files to be uploaded. It would be nice that this R package provides example datasets along with the package so that they can be played in R directly.

 Response: Example negative, positive, and independent datasets are included in the R package under the path “inst/extdata/”.

 5. There is an option named ‘Other’ for ‘analyze clonotypes by (column names in parantheses)’. What is other?

 Response: This option exists to allow users to input a custom column name to analyze by. TCR data can vary in formatting (e.g. some files might describe the amino acid column as “aminoAcid” while others can describe it as “aa”) so this option can help users load different data formats into iCAT.

 6. Please include information of computational time for training and prediction steps for each case.

 Response:

 Use case 1:

 Training time: 2.36 minutes

 Classification time: 30.6 seconds

 Use case 2:

 Training time: 47.81 seconds

 Classification time: 6.83 seconds

 Use case 3:

 Training time: 1.50 minutes

 Classification time: 33.12 seconds

 The manuscript was updated to reflect this information.

 7. Although feature from an option of ‘TCRV-CDR3-TCRJ’ is recommended, please include the performance for other features for each test case which could help readers understand and compare features.

 Response: In general, we found that using the J-gene and V-gene info improves accuracy in our testing, but other features are available in case researchers to believe they might get better results with them. Using the amino acid sequence for the monkeypox data,  1/5 negative samples were identified correctly, while 6/10 positive samples were identified correctly. Using the DNA sequence, 5/5 negative samples were identified correctly and 3/10 positive samples were identified correctly.

 8. What does TCRBV stand for?

 Response: It stands for T cell receptor beta chain variable region.

 9. What does VATS stand for?

 Response: It stood for vaccine-associated target sequences. This is now changed to target-associated receptor sequences (TARSs) for consistency in the manuscript.

 10. A correction constant is not shown in equation (2).

 Response: That is correct We updated the sentence for the equation in the manuscript.

 11. Whole genome sequences can distinguish DENGU from ZIKE with 100% accuracy. For the sentence ‘current laboratory testing cannot distinguish between the two’, are you excluding whole genome sequencing data?

 Response: We are limiting that comment to typical laboratory diagnostics which typically does not include whole-genome sequencing.

 12. Is it possible to make change range of acceptable into something like 1:99 or 1~99 instead of 1 99 to indicate range?

 Response: We reflected this comment and iCAT now allows inputting either a space or a colon to delimit range values.

 13. Does the max p-value change automatically according to the training data provided? Or should it be changed manually based on the selected one by Fisher’s t-test?

 Response: The max p-value can be changed manually.  The purpose is to allow the user to modify the stringency of the diagnostic. This could be done after generating the diagnostic library and the user determines they would prefer more (or less) stringent parameters.

 14. Please revise typo in Figure 1(B)

 Response: Fixed typo in the new manuscript version.

 15. Please revise typo with ‘the minimum samples’

 16. Please revise the following sentences:

 - The ratio of Cv to Cn is determined for each p-value. The p-value with the largest Cv:Cn ratio and offers significant coverage to distinguish (v)accinated from (n)aïve samples was chosen.

 - iCAT was configured to analyze by CDR3 amino acid sequence only

 Response: Sentences were revised in the new manuscript version to make them more clear.

 17. If the value is bigger, we can conclude that the sample is more associated to the the training group. Is this a correct sentence?

 Response: The sentence was revised in the new manuscript version.

 18. From the training data, iCAT correctly classified 32 of 32 naïve samples as ”unexposed” and 58 of 58 vaccinated samples as ”exposed” (100% accuracy). Is this a correct sentence?

 Response: The sentence was revised in the new manuscript version.

# Peer review: Reviewer response for version 1

**DOI:** 10.5256/f1000research.30069.r82160

**Published:** 2021-04-12

**Authors:** Shuo-Wang Qiao, Ying Yao

**Affiliations:** 1 Department of Immunology, Oslo University Hospital, Rikshospitalet, University of Oslo, Oslo, Norway; 2 Department of Immunology, University of Oslo, Oslo, Norway

Diagnostic methods based on the detection of disease-specific or disease-associated T-cell receptor (TCR) sequences are novel approaches whose development is still in its infancy. This paper introduces an intuitive, easy-to-use, software package for the analysis of TCR deep sequencing data with the purpose of grouping data into ‘naïve/healthy’ or ‘diseased/exposed’ groups. The statistical methods used is rather rudimentary, but serve the purpose. It demonstrates remarkably high accuracy in three use cases, including a small dataset of COVID-19. However, the choice of parameter settings and how data is split into training and test datasets, is not well explained. Thus, one is left with a feeling that some over-fitting and over-tuning may have occurred. Nevertheless the shortcomings, overall this software package is first of its kind and will be welcomed by the growing community of scientists working with immune receptors and disease prediction.

**Major comments:**

1. Introduction: Aside from the immensity of TCR diversity, both theoretical and within each individual, by far the largest challenge to identifying signals of T-cell memory to a particular antigen, is the rarity of specific T cells to any given antigen. In circulation during the homeostatic memory phase, this number can be as low as 1 in 100 000 for CD4+ T cells, and may be up to 1% in the CD8 compartment.
2. Operation: Does iCAT support both TCRB and TCRA data, separately, or in the same dataset? Or does it only support TCRB data?
3. Setting (2) during training: at which level of TRBV and TRBJ allelic similarity is the tool tuned? In other words, would human TRBV7-2*01 and TRBV7-2*02 be considered the same, or different? How about human TRBV4-2 and TRBV4-3 that are very similar, especially at the 3’-end where the last 40 amino acids are identical. Many HTS short reads would not be able to distinguish between these two gene segments. 
4. Use Case 1: The authors wrote that they analysed this dataset either by the CDR# amino acid sequence only, or with the V- and J-gene identity in addition. Did these two options behave differently?
5. In the use cases, it is not common to report the accuracy on training dataset. it is not clear how the data was split into the training and testing dataset. More common practice is to do cross validation, and report the averaged precision, sensitivity, maybe F-score, especially for unbalanced samples. For Use case 2 for example, how were the 5 naïve and 10 exposed samples in the test group chosen, randomly? Would one get the same 100% accuracy if another random set of 5 naïve and 10 exposed samples was chosen?
6. In the training tab, the default Max p-value is 0.1. Usually in disease associated studies, a more stringent cut-off p value is recommended. Considering the main purpose of iCAT is for diagnosis, the specificity is not as crucial, 0.1 might be a good choice. Is there any analysis supporting the choice, e.g. in the use cases or on other public available data which antigen specific sequences is known?
7. In the prediction tab, it would be better to provide a measurement of uncertainty in addition to %TARS
8. Some studies such as, Schneider-Hohendorf et al. (2018) ^1^, Britanova et al. (2014) ^2^, and DeWitt et al. (2018) ^3^ suggested that MHC, age and sex also effect the immune receptor repertoire. Incorporating such meta information should be useful, even if in cases where the sample size is too limited for these factors to be modelled in the training, they are better to be randomized in training and testing datasets.
9. How about computing time? How long did it take to do the training, and classification of for instance the use case 2 data?
10. There are substantial public resources including some antigen-specific database and repertoire database. It might be useful to allow loading some commonly used datasets as controls in case the user has limited sample size.

**Minor comments:**

1. Figure legend 1: TCR sequencing of blood samples can be done with, but not limited to, genomic DNA. Many groups also use cDNA.
2. Why does the tip of the iCAT’s tail has an antibody? If this were a TCR analysis tool, would it not be more appropriate with a cartoon drawing of TCR?
3. What is VATS in last paragraph in Page 5, is it the same as TARS?
4. In the library tab, pattern for significant TARS would make more sense, e.g. are they all similar or there were numbers of clusters based on the similarity?

Are the conclusions about the tool and its performance adequately supported by the findings presented in the article?

Yes

Is the rationale for developing the new software tool clearly explained?

Yes

Is the description of the software tool technically sound?

Partly

Are sufficient details of the code, methods and analysis (if applicable) provided to allow replication of the software development and its use by others?

No

Is sufficient information provided to allow interpretation of the expected output datasets and any results generated using the tool?

Yes

Reviewer Expertise:

Immunology, T cells, TCR, single-cell receptor sequencing,

We confirm that we have read this submission and believe that we have an appropriate level of expertise to confirm that it is of an acceptable scientific standard, however we have significant reservations, as outlined above.

## References

[1] : Sex bias in MHC I-associated shaping of the adaptive immune system. *Proceedings of the National Academy of Sciences* .2018;115(9) : 10.1073/pnas.1716146115 2168-2173 10.1073/pnas.1716146115 PMC583468629440397

[2] : Age-related decrease in TCR repertoire diversity measured with deep and normalized sequence profiling. *J Immunol* .2014;192(6) : 10.4049/jimmunol.1302064 2689-98 10.4049/jimmunol.1302064 24510963

[3] : Human T cell receptor occurrence patterns encode immune history, genetic background, and receptor specificity. *eLife* .2018;7: 10.7554/eLife.38358 10.7554/eLife.38358 PMC616209230152754

# Response

**Published:** 2021-06-10

**Authors:** Tae-Hyuk Ahn

**Affiliations:** Saint Louis University, USA

**Major comments:**

 1. Introduction: Aside from the immensity of TCR diversity, both theoretical and within each individual, by far the largest challenge to identifying signals of T-cell memory to a particular antigen, is the rarity of specific T cells to any given antigen. In circulation during the homeostatic memory phase, this number can be as low as 1 in 100 000 for CD4+ T cells, and may be up to 1% in the CD8 compartment.

 Response: We really appreciate all the great and thoughtful comments from the reviewer. As the reviewer commented, the rarity of specific T cells to a specific antigen is also a challenge in addition to the diversity and magnitude of the TCR repertoire. We updated the Introduction from the comment cited a reference for it.

 2. Operation: Does iCAT support both TCRB and TCRA data, separately, or in the same dataset? Or does it only support TCRB data?

 Response: iCAT has been developed and tested to analyze TCR beta chain of the T-cell receptor in the alpha/beta T cells. The column call for the V and J are vGeneName and jGeneName and do not distinguish between alpha and beta. So, as long as the remaining formatting is the same, there is no reason we couldn’t do either TCRA or TCRB.  However, it is not designed to do so in tandem.

 3. Setting (2) during training: at which level of TRBV and TRBJ allelic similarity is the tool tuned? In other words, would human TRBV7-2*01 and TRBV7-2*02 be considered the same, or different? How about human TRBV4-2 and TRBV4-3 that are very similar, especially at the 3’-end where the last 40 amino acids are identical. Many HTS short reads would not be able to distinguish between these two gene segments.

 Response: We do not look that deeply into the different V and J chains. TRBV7-2*01 and TRBV7-2*02 are in the column vMaxResolved or jMaxResolved, which we do not use for the V and J calling. We use the vGeneName and jGeneName, which for this example, both would be called as TRBV7-2. But TRBV4-2 and TRBV4-3 will be called differently as those would be distinguished in the vGeneName column.  You can see this in the data table of the generated library, the selected V and J regions do not include the *01 or *02 details. This is also true when you look in the TSV file.

 4. Use Case 1: The authors wrote that they analysed this dataset either by the CDR# amino acid sequence only, or with the V- and J-gene identity in addition. Did these two options behave differently?

 Response: Using the V- and J-gene identity in addition to the CDR3 amino acid sequence results in significantly higher prediction accuracy in our testing, likely due to identifying more unique markers. Specifically, it results in fewer false positives. For example, in use case 1, using the amino acid sequence only resulted in correctly identifying only 1 / 10 of the pre-vaccination samples, while still correctly identifying 10/10 of the post-vaccination samples.

 5. In the use cases, it is not common to report the accuracy on training dataset. it is not clear how the data was split into the training and testing dataset. More common practice is to do cross validation, and report the averaged precision, sensitivity, maybe F-score, especially for unbalanced samples. For Use case 2 for example, how were the 5 naïve and 10 exposed samples in the test group chosen, randomly? Would one get the same 100% accuracy if another random set of 5 naïve and 10 exposed samples was chosen?

 Response: We agree it is not common to report the accuracy on the training data and it is not as useful as the accuracy on the independent testing data in evaluating a classification tool. However, we chose to include it to demonstrate this feature of the iCAT interface. This feature may be useful as a baseline-check after training in the usual scenarios where the ground truth for the testing data is not available.

 The independent samples in use case 2 were chosen randomly before training and excluded from the training set. We also repeated the test three times after receiving this question using a script to randomly select 15 samples for testing. The accuracy was at a 100% for all the new random tests.

 6. In the training tab, the default Max p-value is 0.1. Usually in disease associated studies, a more stringent cut-off p value is recommended. Considering the main purpose of iCAT is for diagnosis, the specificity is not as crucial, 0.1 might be a good choice. Is there any analysis supporting the choice, e.g. in the use cases or on other public available data which antigen specific sequences is known?

 Response: The max P-value is set at default to 0.1, it can be changed to any value.  We found through our own testing groups that it was a reasonable cut-off (for a default value) as p-values above this tended to generate libraries that were not stringent enough. However, the user can manually alter the p-value to more stringent conditions.  It is worth noting that while the max p-value of the assay may be defaulted at 0.1, iCAT includes a feature for determining the optimal p-value threshold for library generation, which is likely to be well below the max allowed p-value.

 7. In the prediction tab, it would be better to provide a measurement of uncertainty in addition to %TARS

 Response:  This is a great comment and we totally agree that a measurement of uncertainty in addition to %TARS might be useful for iCAT users. We considered an uncertainty metric based on the difference between the probability density in the pre-exposure distribution and the probability density in the post-exposure distribution. This new feature has been implemented and now available on GitHub iCAT and we updated the README screenshot of the prediction tab.

 9. Some studies such as, Schneider-Hohendorf et al. (2018)1, Britanova et al. (2014)2, and DeWitt et al. (2018)3 suggested that MHC, age and sex also effect the immune receptor repertoire. Incorporating such meta information should be useful, even if in cases where the sample size is too limited for these factors to be modelled in the training, they are better to be randomized in training and testing datasets.

 Response: We agree this meta information can provide insightful context for different use cases. This is one of our goals for future versions of iCAT (iCAT 2.0). For example, we are especially excited to study the effect of clustering data based on HLA subtypes. In our opinion, this would be better explored in a separate paper.

 *manuscript modification*10. How about computing time? How long did it take to do the training, and classification of for instance the use case 2 data?

 Response: Following are the results from use case 2.

- Training time: 47.81 seconds
- Classification time: 6.83 seconds

We updated the manuscript to reflect this information.

 11. There are substantial public resources including some antigen-specific database and repertoire database. It might be useful to allow loading some commonly used datasets as controls in case the user has limited sample size.

 Response: Providing commonly used datasets as default controls would be a convenient feature for some use cases. We considered including such datasets with the iCAT software package, but decided against it in the current version due to size consideration. We aimed to create a versatile and easy to use software tool. Using the instructions in this paper and on GitHub, we believe loading public datasets as needed can be performed conveniently by the average iCAT user. We will consider this feature in iCAT 2.0.

**Minor comments:**

 1. Figure legend 1: TCR sequencing of blood samples can be done with, but not limited to, genomic DNA. Many groups also use cDNA.

 Response: Fixed in the new manuscript version.

 2. Why does the tip of the iCAT’s tail has an antibody? If this were a TCR analysis tool, would it not be more appropriate with a cartoon drawing of TCR?

 Response: While it is true that a TCR tail would be more descriptive of our tool, it is our opinion that an antibody makes a more aesthetic cat tail than a TCR. Its shape is also more widely recognizable and can give a general idea to non-specialists about what iCAT deals with at first glance.

 3. What is VATS in last paragraph in Page 5, is it the same as TARS?

 Response: Yes. Fixed in the new manuscript version.

 4. In the library tab, pattern for significant TARS would make more sense, e.g. are they all similar or there were numbers of clusters based on the similarity?

 Response: The current version of iCAT does not perform clustering of the target-associated receptor sequences based on similarity, but this can be developed and incorporated in future versions. We agree that it would be a useful addition for exploratory analyses. iCAT currently focuses on using the sequences for prediction, but provides a starting point for further analyses by allowing the download of those significant sequences and some statistical information about their frequency in the training data.
